# Chitosan–glycerol blended nanofibers for peripheral nerve regeneration applications

**DOI:** 10.1039/d4na01038h

**Published:** 2025-08-12

**Authors:** L. Scaccini, A. Sensini, D. Puppi, M. Gagliardi, L. Moroni, M. Cecchini, P. Wieringa, I. Tonazzini

**Affiliations:** a NEST, Scuola Normale Superiore Piazza San Silvestro 12 Pisa 56127 Italy; b Department of Complex Tissue Regeneration, MERLN Institute for Technology-Inspired Regenerative Medicine, Maastricht University Maastricht the Netherlands p.wieringa@maastrichtuniversity.nl; c Cell Biology-Inspired Tissue Engineering, MERLN Institute for Technology-Inspired Regenerative Medicine, Maastricht University Maastricht the Netherlands; d BIOLab Research Group, Department of Chemistry and Industrial Chemistry, University of Pisa UdR INSTM – Pisa Via G. Moruzzi 13 Pisa 56124 Italy; e Istituto Nanoscienze – CNR, Pisa Piazza San Silvestro 12 Pisa 56127 Italy

## Abstract

Over the last decade, chitosan has drawn increasing attention in the field of regenerative medicine as a natural polymer that is biocompatible, biodegradable, non-immunogenic and widely available. However, while chitosan scaffolds (*e.g.* nanofibers, films, *etc.*) have been developed by multiple methods, the application of these scaffolds has been hampered by the brittle and fragile nature of this material. Chitosan plasticization is possible as a means of improving mechanical properties, with the incorporation of glycerol within solid films having demonstrated its potential to enhance the mechanical properties of chitosan. In this work, we report the first use of plasticized chitosan for the production of nanofibers. We produced aligned glycerol plasticized chitosan nanofibers (Gly–Chi NFs) using electrospinning. Glycerol was used as a plasticizer in order to modulate the mechanical properties of chitosan. We optimized the production process and the glycerol concentration, and measured the surface wettability, FT-IR spectra and thermal properties of the NFs. We obtained continuous, defect-free Gly (2%)–Chi nanofibers with a diameter of 184 ± 6 nm and a high level of anisotropy. These Gly–Chi aligned nanofibers also exhibited biocompatibility with primary Schwann cells *in vitro*, and were effective in guiding their bipolarization, thus suggesting their potential for peripheral nerve regeneration applications.

## Abstract 2

We produced aligned glycerol plasticized chitosan nanofibers with poly-ethylene oxide by electrospinning, with a diameter <200 nm. The anisotropic nanofibers presented an optimal biocompatibility with primary Schwann cells *in vitro*.

## Graphical abstract

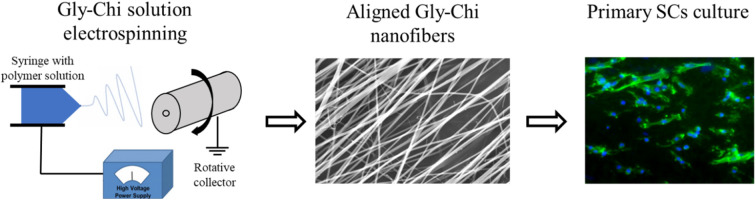

## 1. Introduction

In recent years, the advances in biomaterials research, combined with modern fabrication technologies, led to a rise in the production, testing and use of scaffolds for neural applications. Many different materials and structures have been tested, and today it is accepted that modifying a material to exhibit specific nanostructures could increase the scaffolds' regenerative potential.^1^ In particular, directional nano/microtopographies and nanofibers have been used to functionalize scaffolds and nerve guidance conduits for peripheral nerve regeneration, in order to provide directional topographical guidance to neural cells.^2–4^ This has enabled scientists to harness the natural healing capacity of the peripheral nervous system (PNS). A key player in this process are glial Schwann cells (SCs), myelinating cells of the PNS that start to proliferate and migrate from the proximal stump of the damaged nerve, and provide structural and biochemical support for the subsequent neuronal regrowth.^5,6^ Nanofibers have garnered considerable attention due to their ability to mimic some features of the native extracellular matrix and to provide an optimal substrate for Schwann cell attachment, differentiation, and migration. This is possible because glial cells can perceive and respond to nanometric topographies around them, by a process called mechano-transduction.^7^ Nanometric features are able to modulate glial cell morphology and their migration capability, depending on the size of the topographical structures.^8^ The use of scaffolds with aligned nanofibers is currently under investigation and has already demonstrated the potential to allow for a faster nerve regeneration.^2,9,10^ Moreover, it is emerging how the mechanical properties of a scaffold can also play a pivotal role in determining cell fate, with material stiffness acting as a key regulator of nervous tissue development and regeneration.^11^

In this framework, the search for optimal materials for tissue engineering has led to extensive investigations into natural polymers. This includes chitosan (Chi), which is a biocompatible, biodegradable and non-immunogenic material, produced through the controlled deacetylation of chitin.^12^ Its use in biomedical applications has been approved by the Food and Drug Administration (FDA) and the European Medicines Agency (EMA).^13^ A range of Chi-based scaffolds for tissue engineering have been reported, generated *via* chemical or physical modification, freeze-drying, lyophilization, 3D printing and electrospinning to achieve desired properties and architectures for specific tissue applications.^14,15^

One such fabrication approach that is particularly promising for PNS applications is electrospinning (ES), a well-established technique that relies on an electric field to extrude a polymer/solvent solution from a containing vessel (usually a syringe) to a collector. During the electrospinning process, a charged fluid jet is formed and undergoes extensive stretching and thinning under the effect of the electric field, while the solvent simultaneously evaporates to produce nanofibers deposited onto a grounded collector.^16^ ES-based fibrous scaffolds have been widely reported to be successful at promoting nerve growth, particularly when a high degree of fiber alignment is achieved to effectively direct nerve growth.^17^ While chitosan-based scaffolds have shown promising results for neural tissue engineering applications^18,19^ processing chitosan *via* ES is often a challenge and the final fibrous scaffold is typically fragile and brittle due to the intrinsic mechanical properties of this material.^20^ A widely-used strategy to address this issue is to combine chitosan with other agents, such as polycaprolactone (PCL),^21–23^ poly(hydroxybutyrate) (PHB)^24^ and high molecular weight poly-ethylene oxide (PEO) (600–4000 kDa), which have been tested to improve the outcomes of electrospinning. While many studies confirm that PEO can improve chitosan spinnability (*i.e.* PEO increases the molecular entanglement of the polymer solution), drawbacks remain for this approach. Duan *et al.* produced 1 : 1 Chi-PEO nanofibers, however, when increasing the Chi-to-PEO ratio, low quality or no fibers at all could be produced.^25^ Pakravan *et al.* successfully obtained good quality nanofibers from a Chi-PEO mixture, with a diameter ranging from 140 to 160 nm. However, a temperature of at least 40 °C was needed, as the same process at room temperature led to the formation of beads.^26^ Also, Lemma *et al.* prepared Chi-PEO nanofibers but their overall shape and diameter were affected by the neutralization process, which prevents chitosan nanofibers dissolution by neutralizing the protonated amino groups (–NH_3_^+^) to neutral amines (–NH_2_).^27,28^

To further improve the production and stability of this type of nanofiber, the use of Chi plasticizers can be of interest.^29^ Among the different plasticizers, polyols and glycerol have been the most tested over the years.^30,31^ Glycerol (Gly) has been extensively studied and is known for its ability to lower the stiffness of chitosan-based films. For example, Gonçalves *et al.*^32^ developed chitosan/PVA/glycerol nanofibers for skincare and showed that glycerol, while used to reduce hydrosolubility, actually hinders the electrospinning process by disrupting polymer chain interactions at higher concentrations. However, their work focused on skin applications and required synthetic polymer blending. To date, glycerol's role as a plasticizer in electrospinning pure or primarily chitosan-based aligned nanofibers for neural applications remains unexplored. We previously developed soft Chi microstructured films through the addition of 10% glycerol and demonstrated that they have an impact on neural cells morphology and behaviour. These Gly–Chi blended membranes had a lowered stiffness in comparison to their full chitosan homologues and led to an enhanced migration speed of SCs grown on them.^33^ In addition, glycerol-plasticized chitosan films are also recently reported to have other improved properties such as antibacterial activity, biocompatibility, water vapor permeability, and tensile strength.^34,35^ However, to the best of our present knowledge, no data is available for the use of chitosan plasticizers in electrospinning.

In this work, we developed an original composition of glycerol-plasticized chitosan electrospun nanofibers with an aligned morphology (Gly–Chi NFs). We investigated the morphological, mechanical and thermal characteristics of these nanofibers. Moreover, we tested them with primary rat SCs, with the aim to use them for peripheral nerve regeneration applications.

## 2. Materials and methods

### 2.1 Materials

Chitosan medium molecular weight was purchased from Tokyo Chemical Industry Co. (C2396, Tokyo, Japan). Poly(ethylene oxide) (PEO) was purchased from Sigma Aldrich (average Mv ∼900 000, 189456, St. Louis, Missouri, USA). Glycerol was purchased from Sigma Aldrich (G9012, St. Louis, Missouri, USA).

### 2.2 Preparation of chitosan/PEO/glycerol solution for electrospinning

Chitosan and PEO were dissolved in a 45% glacial acetic acid solution (695092, Sigma Aldrich) and stirred overnight, to create a 4% w/v solution, with a Chi : PEO ratio of 85 : 15. Later, glycerol 0, 2, 5, and 10% in volume was added and the solution stirred overnight.

### 2.3 Nanofibers (NFs) fabrication

The aligned nanofibrous scaffolds were prepared by using an industrial electrospinning apparatus (Fluidnatek® LE-100, Bionicia, Valencia, Spain), equipped with a rotating drum collector (length = 300 mm, diameter = 200 mm) and a humidity/temperature control module. Fibers were electrospun vertically over a nonwoven polyurethane mesh (6691 LL 40 g m^−2^, Lantor B.V.). The previously prepared solution was flowed through a 0.5 mm inner diameter stainless steel needle (Unimed S.A.). Different feed rates, voltages and working distances were used for different glycerol concentrations (Table 1). To obtain aligned nanofibers, the drum collector speed was set at 900 rpm (resulting in a peripheral speed of 9 m s^−1^). During the process, the humidity was set at 25% and the temperature at 25 °C. To prepare individual scaffolds, the nanofibers were punched at the needed dimension, using the nonwoven mesh as a support. Before use, scaffolds were neutralised in a NaOH 125 mM solution for 30 minutes and washed 3 times in water.

**Table 1:** Electrospinning parameters for the chitosan/PEO solutions at increasing concentrations of glycerol

Glycerol concentration (%)	Voltage (kV) (needle; collector)	Feed rate (μL h^−1^)	Working distance (mm)
No glycerol	26; −2	400	140
2%	28; −3	300	125
5%	27; −1.5	400	145
10%	27; −2	500	135

### 2.4 Preparation of chitosan-based membrane by air-drying to serve as controls

The chitosan/PEO/glycerol (2%) solution selected for NFs' electrospinning was cast onto glass surfaces and air-dried. The chitosan used here was the same batch of chitosan used for making electrospun mats. These dried films were neutralized with NaOH 125 mM aqueous solution for 30 minutes, washed three times with deionized water for 5 minutes, and used as control samples.

### 2.5 Chitosan/PEO/glycerol nanofibers imaging

For the imaging, samples were mounted on dedicated stubs and prepared by covering them with a gold coat. NFs meshes were imaged using a Scanning Electron Microscope (SEM) (JSM-IT200, Jeol, Tokyo, Japan), with an accelerating voltage of 10 kV. At least 5 images were acquired for each condition and sample. The distribution of fiber diameters (mean and standard deviation) was measured on the SEM images of about 200 fibers, by means of the image analysis software ImageJ (Ver. 1.54f, NIH, Bethesda, MD, USA).^36^

The orientation of NFs on the surface of scaffolds was investigated *via* the directionality plugin of Fiji (ImageJ).^37^ This approach allowed us to quantify the number of nanofibers within a given angle range from the axis, using a local gradient orientation method, following a previously validated procedure.^38^ This analysis returned a directionality histogram: isotropic images generate a flat histogram, whereas oriented images give a peaked histogram. These histograms were finally fitted by Gaussian curves that returned two parameters, dispersion and directionality (the standard deviation and the centre of the Gaussian curve, respectively), the first representing the degree of orientation of the image, the second representing the direction in which it is oriented (here normalized to the underlying pattern orientation direction). The analysis was performed on five images (magnification = 5000 × *g*) along the pre-neutralization and neutralized scaffold's axis and the results reported as mean ± SD between five images per each region. For the analysis the axial orientation was considered 0° while the transversal one was considered 90°.

### 2.6 Fourier Transform Infrared (FT-IR) spectra analysis

The acquisition of FT-IR spectra was carried out on a Cary 630 FTIR Spectrometer, in the spectral range 400–4000 cm^−1^, with a resolution of 4 cm^−1^. Both the raw materials, control films and nanofibers were tested.

### 2.7 Chitosan/PEO/glycerol nanofibers mechanical testing

The mechanical tests for the NFs were conducted using an ElectroForce 3200 uniaxial testing machine (TA Instruments, New Castle, Delaware, USA), equipped with a 40 N load cell (TA Instruments, New Castle, Delaware, USA). The instrument was controlled using Wint7 software. Samples (high = 40 mm, including the clamping sides of 10 mm each; wide = 10 mm) were mounted in a custom-made non-woven mesh frame (see Fig. 5a) to keep them in position, and mechanically fixed to the clamps. The test was done using an actuator speed of 0.2 mm s^−1^ (strain rate = 1% per s). The thickness of samples was measured using a Trimos Vectra Touch 600 (Swiss Instruments, Mississauga, Ontario, Canada). Five samples for each condition (*i.e.* non-neutralized = NN and neutralized = N) were measured, with a gauge length of 20 mm. The following indicators were calculated: Young modulus (E), yield stress (*σ*_Y_), yield strain (*ε*_Y_), failure force (*F*_F_), failure stress (*σ*_F_), failure strain (*ε*_F_), work to yield (*L*_Y_), Work to Failure (*L*_F_), porosity. After the experiment, each 20 mm gauge length of samples was weighed using a precision balance.

The load displacement curves were converted to stress–strain curves using two different approaches (Fig. 5):^39^

• To describe the macroscopic mechanical behaviour of the specimen, the apparent (a) stress (*σ*_a_) was computed dividing the force by the cross-sectional area measured before the test (*i.e.* calculating also *E*_a_ and *L*_a_).

• To quantify the net mechanical properties, by excluding the contribution of void spaces and only considering the net amount of nanofibers in the samples, the net (*n*) stress was also computed dividing the apparent stresses by the volume fraction (*v*) of the specimens (*i.e.* calculating also *E*_n_ and *L*_n_):
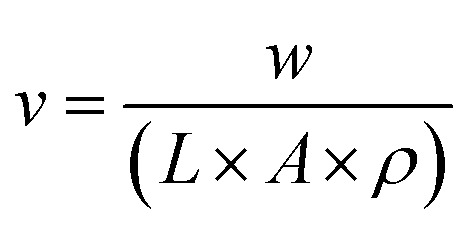
where:

- *w* is the weight of the gauge length of the specimen in *g*.

- *L* is the length of the gauge length of the specimen in mm.

- *A* is the cross-sectional area of the specimen in mm^2^.

- *ρ* is the density of the raw materials in g cm^−3^ (chitosan = 0.38 g cm^−3^, PEO = 1.21 g cm^−3^, glycerol = 1.26 g cm^−3^, equivalent density of the solution = 0.951 g cm^−3^)

The porosity (*P*%) was calculated as follows:_*P*%_ = (1 − *ν*) × 100

### 2.8 Thermal analysis

Thermogravimetric analysis (TGA) was performed on a Q500 instrument (TA Instruments, New Castle, DE, USA). Samples were analysed under a nitrogen flow of 60 mL min^−1^ in the temperature range of 30–700 °C, at a heating rate of 10 °C min^−1^. Maximum temperatures (*T*_max_) were taken as peaks in the first derivative of the TGA thermograms. Differential scanning calorimetry (DSC) analysis was performed on a DSC250 instrument (TA Instruments, New Castle, DE, USA). Samples were analysed in the temperature range of 25–200 °C, with a heating and cooling rate of 10 °C min^−1^, under a nitrogen flow of 50 mL min^−1^. Polymer melting temperature (*T*_g_) was taken at the peak point in the second heating thermograms. Control films and nanofibers, both NN and N scaffolds, were tested.

### 2.9 Primary Schwann cells culture

Primary Schwann cells were extracted from Wistar rat's sciatic nerves following a pre-established protocol.^40^ SCs were cultured in DMEM high glucose, additioned with 4 mM Glutamax, 10% FBS, 1% N2, 20 μg mL^−1^ bovine pituitary extract (BPE), 5 μM forskolin and 1% pen/strep. Cells were cultured at 37 °C and with a CO_2_ concentration of 5%.

Cells were seeded on the Gly (2%)–Chi/PEO NFs at a seeding density of 50 000 cells per cm^−2^ and let grow for 6 ± 1 day, changing the media every two days. Glass coverslips were also used as standard control substrates.

### 2.10 Immunostaining

SCs were grown for 6 ± 1 day on Gly (2%)–Chi NFs, then fixed for 30 minutes in 4% paraformaldehyde in PBS at room temperature (RT), and washed 3 times with PBS. Cells were treated with blocking buffer (3% BSA, 5% goat serum, 0.05% Tween in PBS) for 2 h at RT and then stained with phalloidin-Alexa750 (ThermoFisher; A30105, 1 : 50) in blocking buffer overnight at 4 °C, to stain actin fibers (F-actin). Samples were then washed, incubated with 4′,6-diamidino-2-phenylindole (DAPI) for 15 minutes and then washed in PBS. Eventually samples were mounted using Dako fluorescence mounting medium (Agilent Technologies, S302380-2).

### 2.11 Cell imaging and morphological analysis

Images were acquired using either a Nikon Eclipse Ti-E inverted epifluorescence microscope (Nikon, Tokyo, Japan) equipped with a 20× air Nikon objective (NA 0.75, Plan Apo λ) or a Leica Stellaris (Leica Microsystems, Wetzlar, Germany) equipped with a 20× air Leica objective. Each reported image was acquired as a *Z*-series and subsequently processed using ImageJ software into a single image using the “Extended Depth of Field” plugin (developed by the Biomedical Engineering Group at EPFL).

To assess the proliferation rate on Gly–Chi NFs, the nuclei were quantified on different samples after 6 days, as in ref. 41. The images of the F-actin were used to evaluate morphological descriptors of cells by ImageJ software (NIH), as in ref. 8. The cell contours were drawn using the “Polygon Selection” tool and quantified using the command “Measure”, with the options “Area”, “Shape Descriptors” and “Fit Ellipse”. The aspect ratio was calculated as a ratio between the major axis and the minor axis of cells. A SCs bi-polarization index was calculated measuring the percentage of bi-polarized SCs (using the previously drawn cells' ROI) *versus* the total number of cells; here protrusions with length <10 μm were excluded from this analysis, similarly to ref. 42.

### 2.12 Statistical analysis

All the experiments were repeated at least three times independently for each condition. Data are reported as the average value ± the standard error of the mean (mean ± SEM), unless differently stated. The mean values obtained in each repeated experiment were assumed to be normally distributed around the true mean. To compare two conditions (NN and N scaffolds), unpaired Student's *t*-test was used. Statistical significance refers to results where *P* < 0.05 was obtained.

## 3. Results

### 3.1 The role of glycerol concentration on the production process of the chitosan–glycerol blended nanofibers

**Fig. 1:**
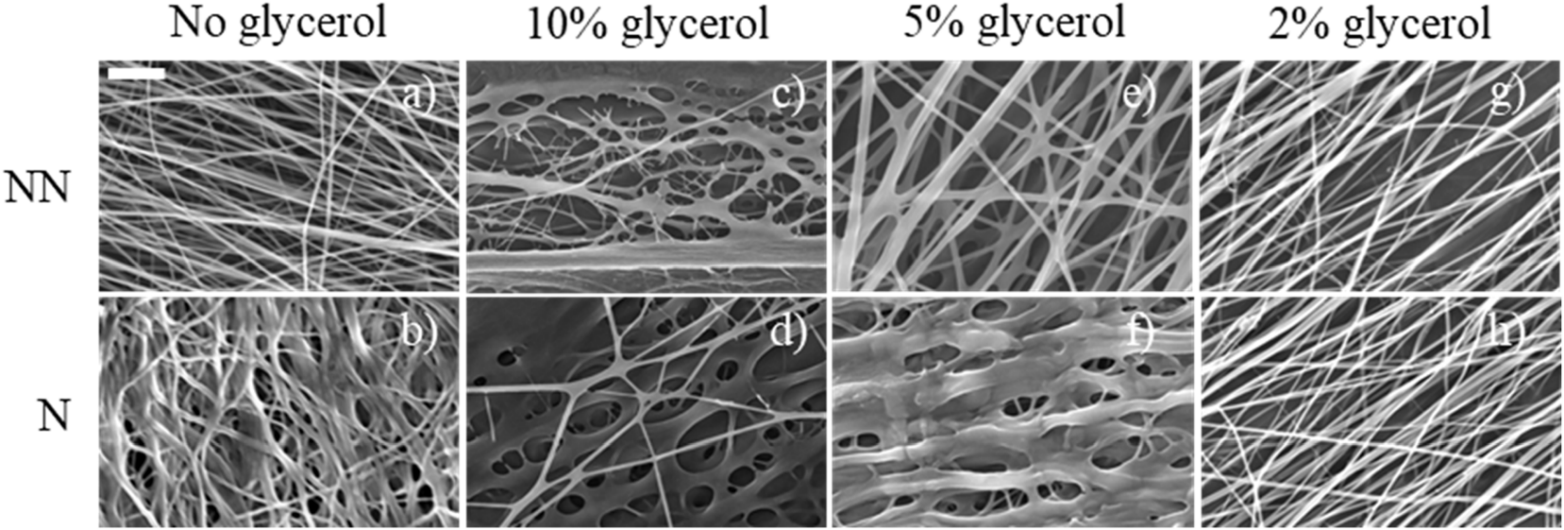
(a–h) SEM images of Gly–Chi NFs at 10.000-fold magnification for the different concentrations of glycerol tested (columns), before (NN) and after (N) the neutralization process (rows). Scale bar = 20 μm.

In order to produce aligned chitosan–glycerol blended nanofibers (Gly–Chi NFs), we started by creating the base solution of Chi : PEO. The PEO was added at low concentration (15% in weight out of the total polymer concentration), as a fiber forming agent that supports the creation of the NFs. Glycerol concentrations of 10, 5 and 2% v/v were tested. To produce a proper alignment of the nanofibers, the different solutions were electrospun on a high-speed rotating drum collector. Gly–Chi NFs were imaged by SEM, both at NN and N stage (Fig. 1). We also effectively obtained Chi/PEO NFs, without glycerol. However, they were extremely rigid and fragile and could not be detached from the support substrate upon which they were electrospun.

The Gly concentration affected the NFs morphology, with 10% Gly concentration resulting in no NFs being properly formed (Fig. 1c and d). At 5% Gly concentration, NFs appeared homogeneous, continuous and without the presence of beads: however, after the neutralization process, these 5% Gly–Chi NFs lost their structural integrity (Fig. 1e and f). Finally, the use of 2% Gly concentration led to the formation of continuous, homogeneous, smooth and beads-free NFs that were able to retain their structure after the neutralization process (Fig. 1g and h). In terms of size, these 2% Gly–Chi NFs had a diameter of 184 ± 2 nm.

To assess the directionality and alignment of the electrospun Gly–Chi NFs, we performed a local gradient components analysis on the SEM images, observing a preferential axial alignment of the NFs with a Gaussian-like distribution (Table 2), independently from the concentration of Gly. The dispersion value was also measured, giving similar results between the different conditions, and laying between 9.0° and 14.2°.

**Table 2:** Average diameter, alignment angle and dispersion for Gly–Chi NFs, non-neutralized (NN) and after neutralization (N), at the different concentrations of glycerol. Data = mean ± SEM, *n* = 3

Glycerol concentration (%)	Condition	Diameter (nm)	Alignment angle (°)	Dispersion (°)
No glycerol	NN	181 ± 45	3.4 ± 2.7	9.0 ± 4.0
	N	No fibers	No fibers	No fibers
10	NN	No fibers	No fibers	No fibers
	N	No fibers	No fibers	No fibers
5	NN	315 ± 115	2.6 ± 2.2	13.3 ± 9.1
	N	No fibers	No fibers	No fibers
2	NN	184 ± 2	2.5 ± 0.6	9.5 ± 1.2
	N	179 ± 3	4.6 ± 0.9	14.2 ± 1.3

Because of the optimal physical features, enhanced stability after neutralization and the consistent diameter observed for the 2% Gly–Chi NFs, we decided to proceed with them for further experiments.

**Fig. 2:**
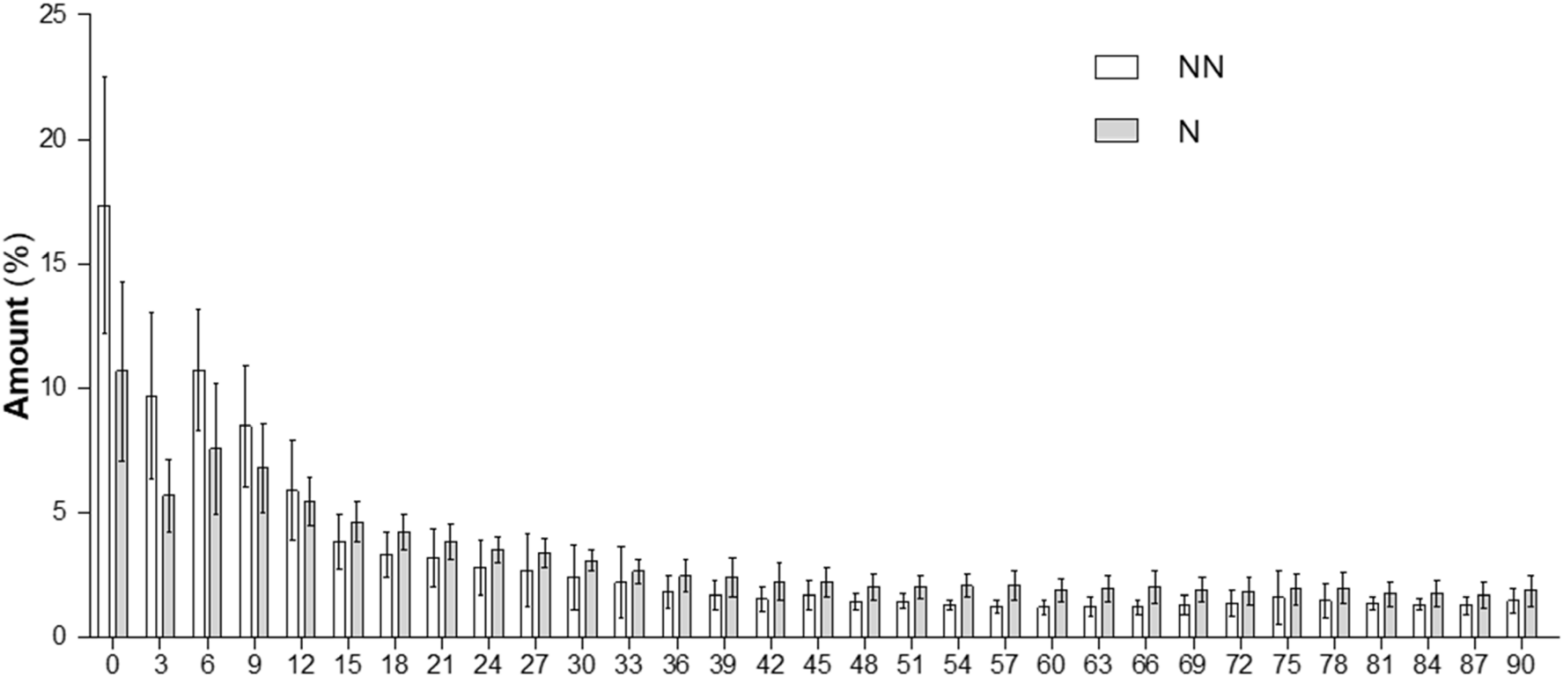
Directionality analysis on Chi–Gly NFs, pre- (NN) and post-neutralization (N). An angle of 0° means that the NFs were aligned with the longitudinal axis of the specimens, while an angle of 90° means that NFs were perpendicular to the longitudinal axis of the specimens. Data = mean ± SEM (*n* = 3).

We then analysed the angular distribution of NFs alignment (Fig. 2). We observed that ≈56% of the NN-NFs lay between 0 and 15° in respect to the main axis direction of the fibers mat. This value tended to slightly decrease after the neutralization process, resulting in approximately 40% of Gly–Chi NFs with an angle between 0 and 15°. Furthermore, in both cases, less than ≈12% of the NFs were perpendicular to the main axis (*i.e.* deviated more than 75° from the main axis of the NFs). 2% Gly–Chi NFs showed, both pre and post neutralization, a preferential axial orientation with a progressive dispersion in the different range of angles up to 90°. After the neutralization, 2% Gly–Chi NFs tend to reduce their anisotropy, suggesting that this process can be caused by a progressive shrinkage of the NFs due to the loss of PEO during the neutralization process (Table 3).

**Table 3:** DSC and TGA data for 2% Gly–Chi films and nanofibers, non-neutralized (NN) and neutralized (N)

Sample	Condition	DSC				TGA			
		First heating		Cooling	Second heating	Water loss	Decomposition		
		*T*_m_ (°C)	Dehyd (°C)	*T*_c_ (°C)	*T*_m_ (°C)	Peak (°C)	*T*_onset_ (°C)	Peak 1 (°C)	Peak 2 (°C)
Film	NN	—	110	—	—	122	209	245	384
	N	—	108	—	—	—	220	261	402
Nanofibers	NN	48	104	33	58	142	190	241	283
	N	—	103	—	—	—	170	257	—

### 3.2 Fourier-Transformed Infrared (FT-IR) spectra analysis

**Fig. 3:**
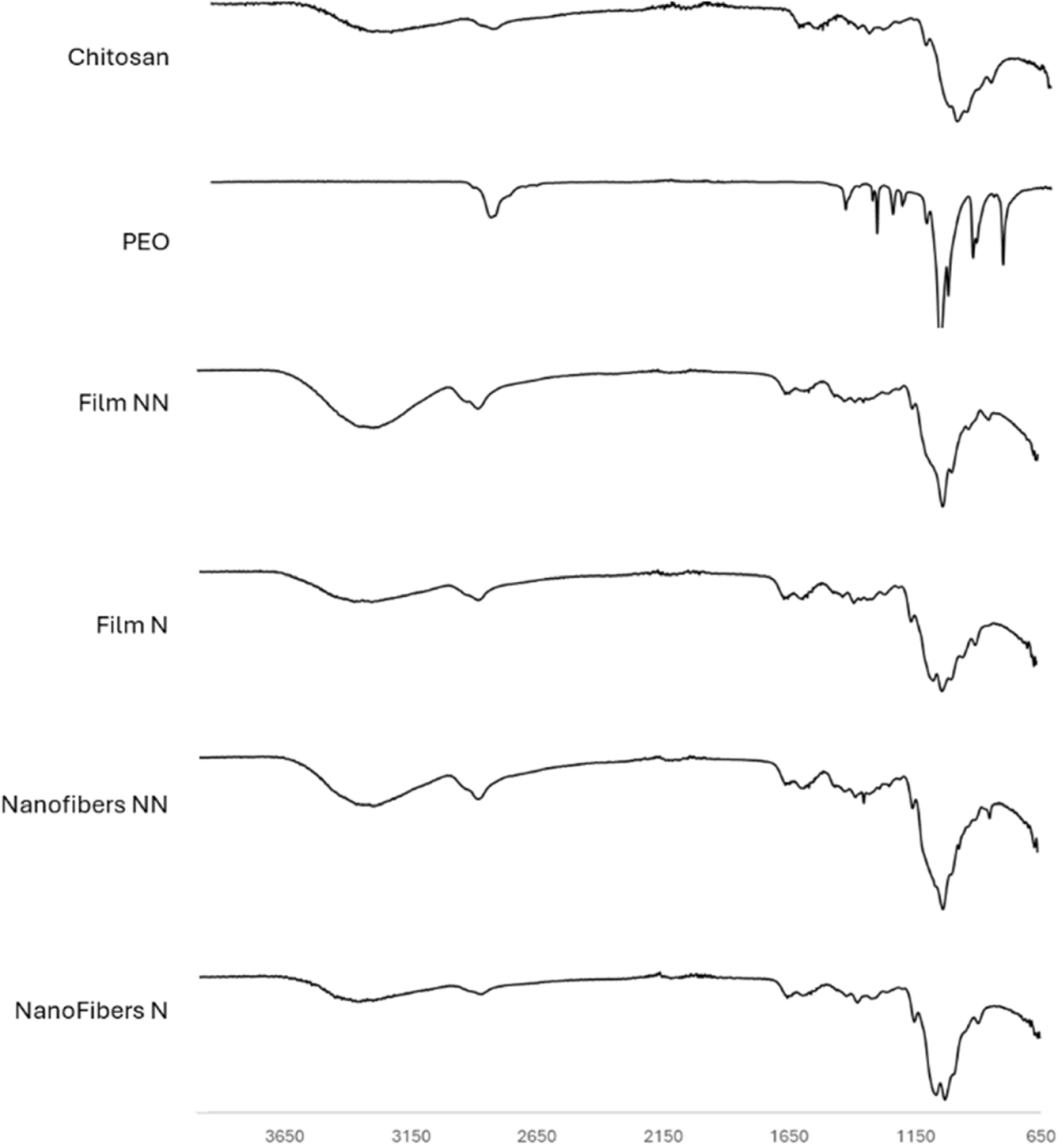
FT-IR spectra for raw materials (chitosan, PEO), Gly–Chi control films (non-neutralized, NN, and neutralized, N) and Gly–Chi NFs (NN and N).

In order to study the composition and functional groups of the Gly–Chi NFs before and after the neutralization step, Fourier Transform Infrared spectroscopy (FTIR) was performed (Fig. 3). In Gly–Chi NFs, a broad band at 3100–3600 cm^−1^ was assigned to N–H and O–H stretching of the polysaccharide molecules and of Gly. A peak at 2900 cm^−1^ was assigned to C–H bonds stretch, typical of chitosan and PEO. A small peak at around 1700 cm^−1^ was attributed to C=O stretching signal of chitosan. At around 1650 cm^−1^ the N–H bending, from chitosan molecules, can be seen. The large peak at around 1000 cm^−1^ was attributed to chitosan's and PEO's C–O bonds stretching. These peaks in the structure of the Gly–Chi NFs indicate that PEO and glycerol are present inside the structure of the pre-neutralization condition. However, after the neutralization, the characteristic peaks of PEO at 2900 cm^−1^ and 1000 cm^−1^ were weaker in intensity, indicating a reduction of the presence of PEO. This is in good agreement with the water-soluble nature of PEO. This data is also confirmed in the control films, in which all the characteristic peaks from PEO and glycerol are present.

### 3.3 Thermal properties

**Fig. 4:**
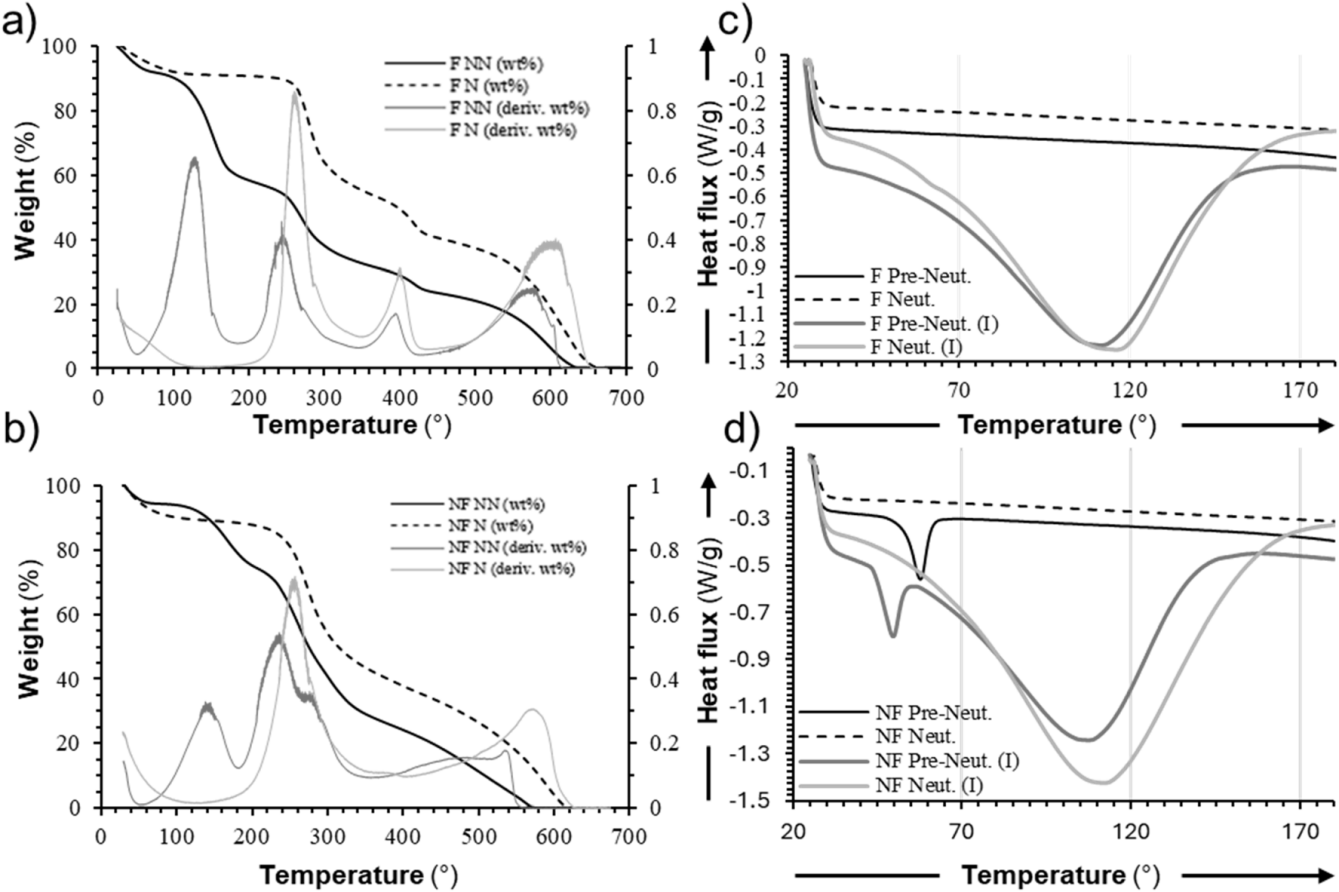
Thermal analysis. (a and b) Representative TGA curves for 2% Gly–Chi (a) control films and (b) NFs. Solid lines represent pre-neutralization samples and dotted lines represent post-neutralization samples. In grey is the first derivative of the relevant solid curves, in light grey is the first derivative of the relevant dotted lines. (c and d) Representative DSC curves for 2% Gly–Chi (c) control films and (d) NFs. Solid lines represent non-neutralized (NN) samples and dotted lines represent neutralized (N) samples.

We performed differential scanning calorimetry (DSC) and thermogravimetric analysis (TGA) to further characterize the composition and properties of our 2% Gly–Chi NFs, using films of the same composition as a control. The TGA analysis of the control 2% Gly–Chi films (Fig. 4a), at temperatures between 20 °C and 800 °C, showed three main mass-loss events for the pre-neutralization samples: the first one, starting at 110 °C and peaking at 150 °C is related to the loss of water and other volatile components; the second one, starting at around 260 °C and peaking at 280 °C, was related to the chitosan decomposition; lastly, the third event, starting at around 400 °C and peaking at 410 °C described the PEO decomposition. After 520 °C the material progressively lost all of its mass. After the neutralization process, the same events were measured, except for the first water evaporation and a general lessening of the peak related to the PEO decomposition. TGA analysis of the Gly–Chi NFs (Fig. 4b) showed a similar qualitative behaviour, with a slightly lower stability at high temperatures than what was observed in films. This is generally attributed to the significantly higher surface area-to-volume ratio of nanofibers, which enhances their exposure to heat and oxygen, thereby accelerating thermal degradation.^43^

DSC analysis results representing the first and second-heating curves of the samples, are shown in Fig. 4c and d. Chitosan, like some other polysaccharides, suffers thermal degradation without melting, especially under extreme temperatures.^25^ During the first scan, a broad endothermic peak at around 100 °C can be seen: this is attributed to the water evaporation from the sample. During the second scan, an endothermic peak was observed at 58 °C in Gly–Chi NFs before the neutralization, attributed to the melting of PEO. The same event was not present in NFs samples after the neutralization process. These results indicate that PEO presence is effectively detected in 2% Gly–Chi NFs only before the neutralization step.

### 3.4 Mechanical properties

**Fig. 5:**
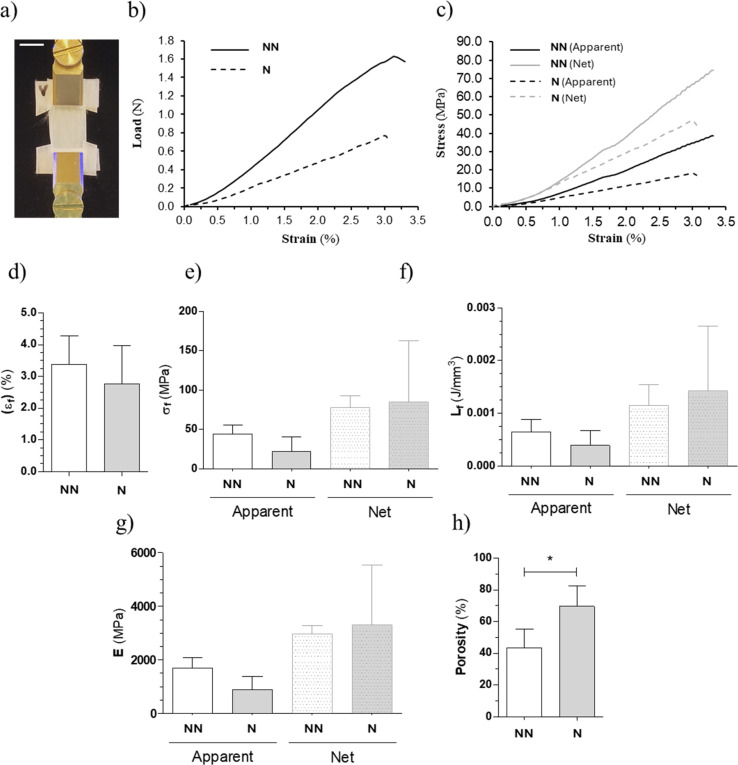
Mechanical tensile tests of the Gly–Chi non-neutralized (NN) and neutralized (N) samples. (a) Setup for the measurements. Scale bar = 10 mm (b) example of load-strain curves of a typical Gly–Chi NFs sample. (c) Example of the apparent (solid lines) and net (dashed lines) stress–strain curves of a typical Gly–Chi NFs sample. (d–h) Comparison of the mechanical properties between non-neutralized (NN) (left) and neutralized (N) Gly–Chi NFs samples (right): (d) failure strain (*ε*_F_), (e) failure stress (*σ*_F_), (f) work to failure (*L*_F_) and (g) Elastic modulus (apparent = solid bars; net = dashed bars); (h) porosity. Data = mean ± SD. The statistical significance of differences is indicated: *p < 0.05, Student's *t*-test, *n* = 4.

In order to measure the mechanical properties of the 2% Gly–Chi NFs, we performed uniaxial tensile testing (Fig. 5). The strain rate of 1% per s was chosen to simulate a quasi-static biological task.^44^ The NFs mats were held in position by a surrounding framing structure, made of a non-woven polyurethane mesh that acted as a support (Fig. 5a). In general, both the NN- and N-NFs mats presented a nonlinear toe region up to around 1.5% of strain (Fig. 5b and c), caused by the progressive recovery of the nanofibers from their wavy resting state. After that, we observed a linear region and an apparent failure stress for the NN samples of *σ*_NNaf_ = 44 ± 10 MPa and of *σ*_Naf_ = 22 ± 16 MPa for the N samples, with equivalent net failure stress of *σ*_NNnf_ = 78 ± 12 MPa and *σ*_Nnf_ = 84 ± 67 MPa for the NN samples and N samples, respectively. The failure strain of NN samples was *ε*_NNf_ = 3.38 ± 0.77% while for N samples it was *ε*_Nf_ = 2.77 ± 1.04%. The apparent work to failure for the NN samples was *L*_NNaf_ = 0.0006 ± 0.0002 J mm^−3^ and for the N ones *L*_Naf_ = 0.0004 ± 0.0002 J mm^−3^ (with net work to failure values of *L*_NNnf_ = 0.0011 ± 0.0003 J mm^−3^ and *L*_Nnf_ = 0.0014 ± 0.0011 J mm^−3^ for the NN samples and N samples, respectively). The resulting apparent Young's modulus was *E*_NNa_ = 1686 ± 341 MPa for the NN samples and *E*_Na_ = 891 ± 428 MPa for the N samples (*E*_NNn_ = 2982 ± 256 MPa; *E*_Nn_ = 3310 ± 1935 MPa). Overall, all net properties were 2 to 3 times higher than the apparent ones because only the NF contribution was taken into account and the void space was excluded from the calculations.

### 3.5 Schwann cell growth

**Fig. 6:**
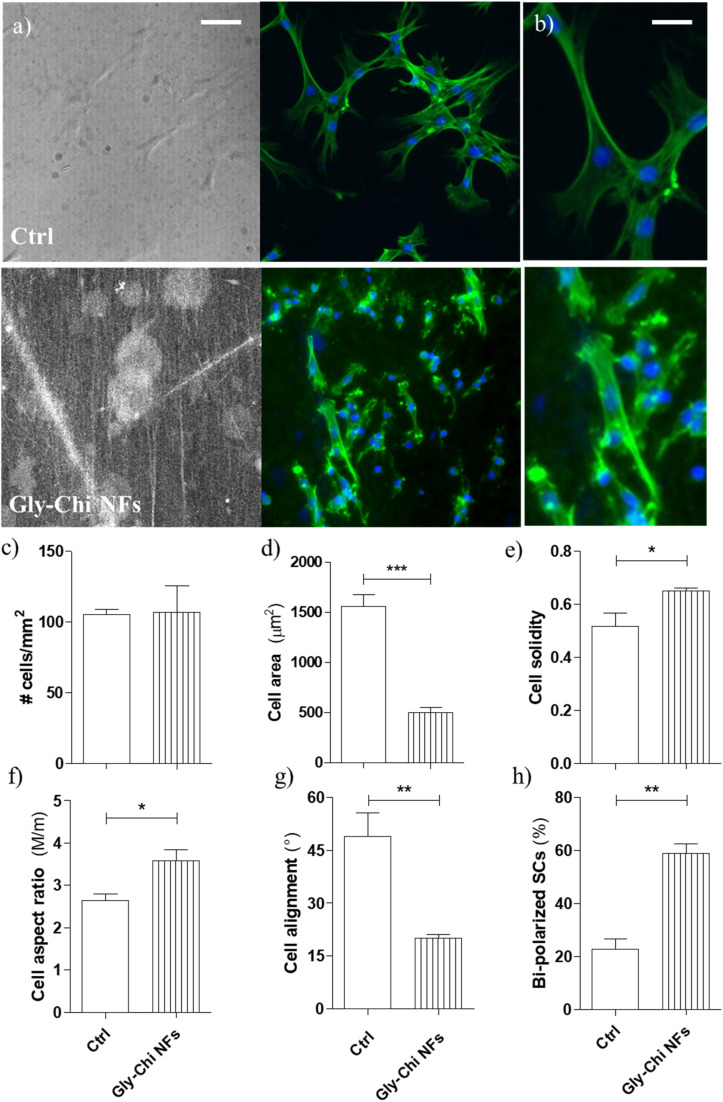
Primary SCs cultures on 2% Gly–Chi NFs. (a) Confocal images of SCs cultured on flat glass coverslips (upper row) and on Gly–Chi NFs (lower row). From left to right: bright field (BF), nuclei (blue), F-actin (green), merged channels. Scale bar = 100 μm. (b) Magnification of the merged channels. Scale bar = 30 μm. (c) Total number of cells in a mm^2^ of sample. (d–h) SCs' morphological characterization. The single-cell morphological analysis was performed by manually tracking each cell shape (on actin fiber images) and measuring: (d) cell area; (e) cell solidity; (f) cell aspect ratio (*i.e.* the ratio of the length of the major axis M to the minor axis m of the best-fitted ellipse on the drawn cell shape); (g) cell alignment angle (°; the angle between the cell major axis and the main axes of the pattern; a random direction was chosen for the glass coverslips); (h) cell bipolarity. *p < 0.05, **p < 0.001, ***p < 0.0001, Student's *t*-test. Data = mean ± SEM, *n* ≥ 3.

To assess the biocompatibility of 2% Gly–Chi NFs, and their potential use in nerve tissue engineering, we cultured primary SCs on our aligned 2% Gly–Chi NFs, up to 1 week. SCs adhered and grew well on 2% Gly–Chi NFs, similarly to those grown on standard glass coverslips as a control (Fig. 6a). The total number of SCs after 1 week in culture was similar on the NFs and the control substrates, with an average value of 107 ± 19 cells mm^−2^ on 2% Gly–Chi NFs and 105 ± 4 cells mm^−2^ on glass coverslips. We then analysed SC cell morphology, by measuring various shape descriptors. The mean cell area of SCs cultivated on these NFs was 501 ± 48 μm^2^, significantly smaller compared to SCs on glass coverslips (1560 ± 122 μm^2^; *p* < 0.001, Student's *t*-test; Fig. 6c). SCs' solidity (*i.e.* calculated as the ratio between cell area to the convex hull area encompassing the cell) was significantly enhanced on 2% Gly–Chi NFs in comparison to the flat isotropic control surface (*p* < 0.05, Student's *t*-test; Fig. 6d), thus suggesting that cells have a more regular protrusion pattern.

Primary SCs aligned and polarized in the direction of NFs, with SCs on 2% Gly–Chi NFs exhibiting a polarized morphology with a significant increase in aspect ratio compared to flat control substrates (*p* < 0.05 2% Gly–Chi NFs *vs.* glass, Student's *t*-test; Fig. 6e). The cell alignment angle with respect to the average direction of the NFs was 20.2 ± 1.0° (*p* < 0.01 2% Gly–Chi NFs *vs.* glass, Student's *t*-test; random distribution on glass was 49.0 ± 6.6°; Fig. 6f). Moreover, SCs cultivated on 2% Gly–Chi NFs resulted in highly bi-polarized (*p* < 0.01 *vs.* glass substrate, Student's *t*-test; Fig. 6g), suggesting that these NFs promote the typical SCs' bipolar differentiation phenotype.

Overall, these data indicate that 2% Gly–Chi NFs support the growth of primary SCs, with our nanofibers exerting an active control over SCs growth, bipolarized morphology, and overall alignment along the general NF orientation.

## 4. Discussion

In this work, we present a method for the fabrication of chitosan nanofibers plasticized with glycerol. We obtained oriented, continuous, beads-free nanofibers, with optimal thermal and mechanical properties, by using PEO as fibers forming agent and a 2% glycerol amount. Our 2% Gly–Chi NFs were tested with primary Schwann cells and resulted biocompatible and able to direct their cytoskeletal organization, towards the NFs' direction.

To the best of our knowledge, this is the first example of using plasticized Chi in combination with PEO for the production of nanofibers. We exploited electrospinning as a method of production: this technique is well established in literature, with a high level of reproducibility, rapid production, relatively low cost, and amenable to a wide range of customization *via* optimization of fabrication parameters.

The electrospinning of pure Chi is difficult to achieve and often requires additives and toxic solvents. Here, we used a limited amount of PEO as an NF forming agent, thus obtaining continuous and beads-free NFs. The plasticizing effect of glycerol depends on the ability of this molecule to lower the interactions between adjacent polymer chains, which also lowers the viscosity of the polymer solution. Because of this, the addition of Gly required further optimization of the spinning parameters. To obtain aligned Gly–Chi NFs, we used a rotating drum as a collector with a diameter of 20 cm and a rotation speed of 900 rpm, which resulted in an equivalent speed of 9 m s^−1^ of the collector surface. It is known from literature that, to obtain an effective alignment of NFs, an average collector speed of at least 8 m s^−1^ should be reached.^45^ In line with this, we obtained aligned NFs with a low level of angular dispersion. Importantly, the dimensionality of NFs we obtained is in the same magnitude as the dimensionality of extracellular matrix signals found within a peripheral nerve.^46^

One other study by Gonçalves *et al.* describes the electrospinning of chitosan in combination with glycerol,^32^ using instead a Chi/PVA solution with a comparatively lower amount of Gly (0.5 g for 20 + 20 g Chi-PVA). They report the successful production of nanofibers, however NF formation was hindered by the presence of glycerol.^32^ In our system, instead, glycerol played an important role in stabilizing the structure of nanofibers and their handling for further testing. Although being similar, the composition of their electrospinning solution differed by using PVA as a fiber-forming agent instead of PEO, which can be the basis for the different observed behaviour.

Our main motivation in adding glycerol to the chitosan solution was to improve the mechanical properties of our chitosan-based scaffolds, with the aim to have softer scaffolds that more closely approximated nerve tissues. We inadvertently discovered that the addition of glycerol, particularly at relatively low concentration, led to the production of chitosan-based NFs that were both more mechanically robust and, therefore, handleable. A concentration higher than 2% resulted in low-quality NFs, which then resulted spread and melted one to another. This has to do with the plasticizing effect of Gly on Chi: a higher Gly concentration affects the coherence of the resulting material, which was insufficient to retain the NF shape. On the other hand, the absence of Gly resulted in the NFs' extreme rigidity and brittleness. This phenomenon is in agreement with other works in this field and is probably related to the loss of PEO during the neutralization step. This loss of PEO was confirmed by our FT-IR experiments and thermal analysis and is likely because this molecule is water-soluble. The addition of the plasticizer, glycerol, helped in NF morphology retention, also after the neutralization process. By optimizing the amount of Gly, we were able to achieve efficient chitosan NF production, an important step toward fully exploiting Chi NFs for regenerative medicine applications.

The retention of the NF morphology in presence of glycerol could be attributed to the formation of hydrogen bonding with Chi and PEO, thus blocking the entry of water. Glycerol would therefore compensate for loss of ionic interactions in nanofibers after neutralization, which would maintain the structural integrity of the fibers. Further, glycerol itself would act as a placeholder for water, therefore blocking the ingress of water molecules into the fibers to prevent swelling and maintain overall structural stability.

Analysis of the thermal properties of our 2% Gly–Chi NFs indicated suitability of these materials for biomedical applications, with all the recorded thermal degradation events occurring at temperatures higher than found in the body (*i.e.* 37 °C). The higher hydration that was detected in samples that had not undergone the neutralization process is probably due to the presence of intrinsic water, which is lost through evaporation after this process. Therefore, this can explain the absence of a weight loss event at 150 °C observed in neutralized samples.

The NF scaffold porosity (*P*%) observed in N samples was significantly higher compared to NN samples (*P*%_NN_ = 43 ± 6% *vs. P*%_N_ = 70 ± 6%; *p* < 0.05, Student's *t*-test). This was expected, since the neutralization step leads to a shrinking of the nanofibers, increasing the amount of empty space inside the samples. This process also explains the general increase in the ratio between net and apparent properties after the neutralization step. The mechanical properties of our 2% Gly–Chi nanofiber scaffolds have been evaluated and compared with those of native human peripheral nerves, to assess their suitability for neural tissue engineering. Human peripheral nerves typically exhibit a Young's modulus ranging from 0.45 to 0.8 MPa, reflecting their soft nature.^47,48^ While our scaffolds demonstrated a Young's modulus higher than 0.8 MPa when tested in the dehydrated state, this value is expected to decrease significantly under physiological (hydrated) conditions and, therefore, better approximate the viscoelastic properties of fully hydrated native nerve fibers. Similarly, our dry scaffolds showed a failure strain (*ε*_F_) around 10%, which, although lower than the 30–40% strain at failure reported for fresh human ulnar nerves or rodents' sciatic nerves,^47,49^ still falls within a functional range for supporting cellular responses (*e.g.* Schwann cell migration, axonal guidance).

For our final intended application, it is also not a strict requirement that our nanofibers replicate the exact mechanical feature of nerve tissues. Instead, what must be provided is a sufficiently compliant, supportive, and biofunctional environment for neural cells: overall, our aim is to promote nerve regeneration without inducing a mechanical mismatch at the repair site. Thus, while further refinement and testing in hydrated conditions are necessary, the current mechanical profile of our scaffolds indicates promising compatibility with the mechanical demands of peripheral nerve repair.

Finally, our 2% Gly–Chi NFs were able to sustain the growth and proliferation of primary Schwann cells, even without the application of a specific protein coating. These aligned NFs effectively induced cell alignment and bipolarization, likely thanks to their dimension range (≈200 nm) is in the same range of that of unmyelinated PNS axons.^50^ An effective alignment of Schwann cells has been demonstrated to be a precursor for their controlled migration.^51^ This oriented migration can be of fundamental importance for the final performances of scaffolds for peripheral nerve regeneration, as it can help for a correct repair of a severed nerve and lower the regeneration time. Moreover, the possibility to control *via* ES the production of scaffolds made completely of biocompatible and biodegradable materials (*i.e.* both Chi and Gly) presents great advantages. While a more detailed investigation is needed to fully assess the regenerative ability over our oriented 2% Gly–Chi NFs, this work represents a first step towards the use of plasticized chitosan nanofibers to support more effective peripheral nerve regeneration.

## 5. Conclusion

In this work, we show that glycerol-plasticized chitosan NFs can be developed by electrospinning. Our nanofibers are continuous, beads-free, with a diameter of around 180 nm, stable after the neutralization process and can be aligned through the use of a rotative collector. The retention of their shape is due to the presence of the Gly plasticizer. Moreover, these glycerol-chitosan based nanofibers have good thermal and mechanical properties. All these features make them good candidates for future use as scaffolds in nerve regenerative medicine. Herein, we highlight the biocompatibility of our NFs with Schwann cells and their ability to guide and bipolarize these cells. This represents an initial step toward the development of mechanically-compliant and clinically relevant nerve scaffolds. While additional studies are needed to assess the scaffolds' degradation behavior and functional performance *in vivo*, our results warrant this further investigation by providing evidence of the use of plasticized chitosan nanofibers for peripheral nerve regeneration.

## Conflicts of interest

There are no conflicts to declare.

## Data Availability

Data are going to be available at Zenodo at: https://doi.org/10.5281/zenodo.14161233.
